# Supplementary material for: SnoRNA signatures in cartilage ageing and osteoarthritis
Source: Sci Rep. 2020 Jun 30;10:10641. doi: 10.1038/s41598-020-67446-z (PMC7326970; doi:10.1038/s41598-020-67446-z)

**Supplementary File 6** Topographical snoRNA gene expression in non-OA cartilage. RNA extracted from equine metacarpophalangeal joints; low load area was lateral condylar area and high load area was medial condylar area n=5. SnoRNA gene expression relative to U6 and protein coding genes to GAPDH. Data represents mean ± standard error of mean. Statistical analyses undertaken following normality testing with a Mann Whitney Test.


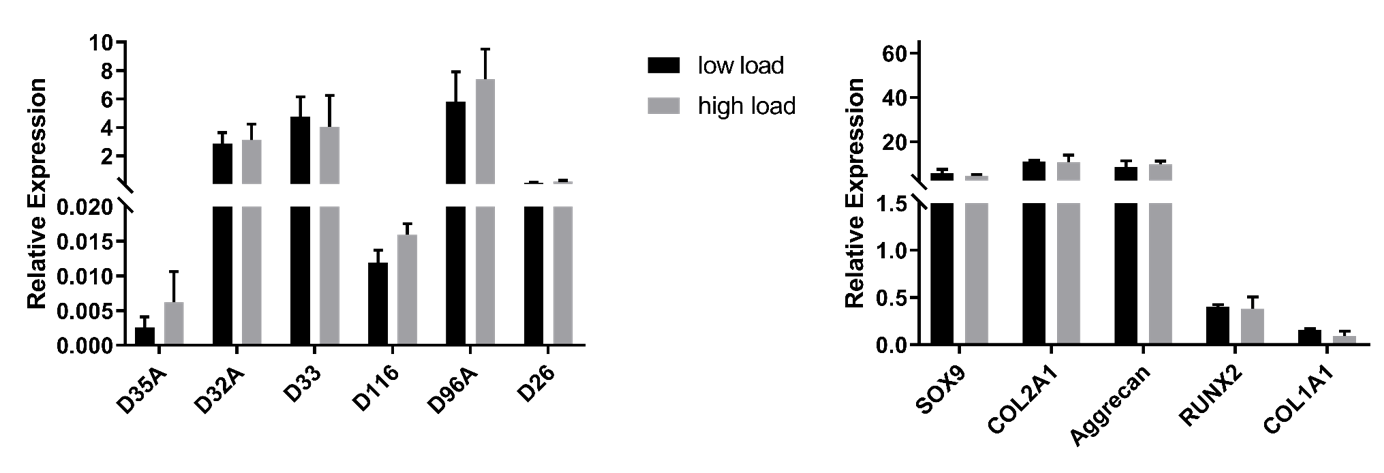

Supplement: Supplementary file 6 — Supplementary file6 [file 41598_2020_67446_MOESM6_ESM.docx]
