# Supplementary material for: SnoRNA signatures in cartilage ageing and osteoarthritis
Source: Sci Rep. 2020 Jun 30;10:10641. doi: 10.1038/s41598-020-67446-z (PMC7326970; doi:10.1038/s41598-020-67446-z)

Supplementary File 7. Expression of mRNA for indicated genes following A. IL-1β treatment or B. OA synovial fluid treatment measured using qRT-PCR. Treatment of non-OA HAC with IL-1β (10ng/ml) for 24 hours or with 20% OA synovial fluid (SF) (derived from a pool of ten donors) for 24 hours. Gene expression changes were measured using 2^-ΔCT expression relative to cyclophilin. Data represents the mean + standard error mean, P values indicated as follows; p<0.05 *, p<0.01 **. Within the graphs black represents control and grey treatments.


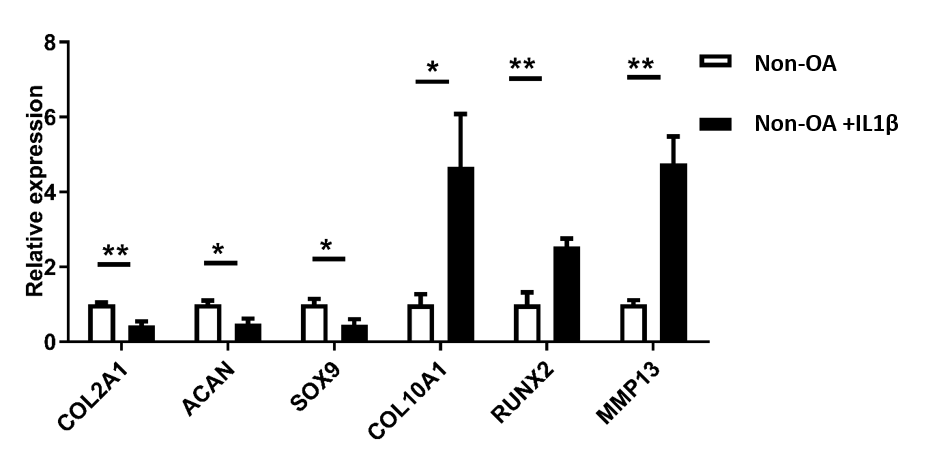


B.


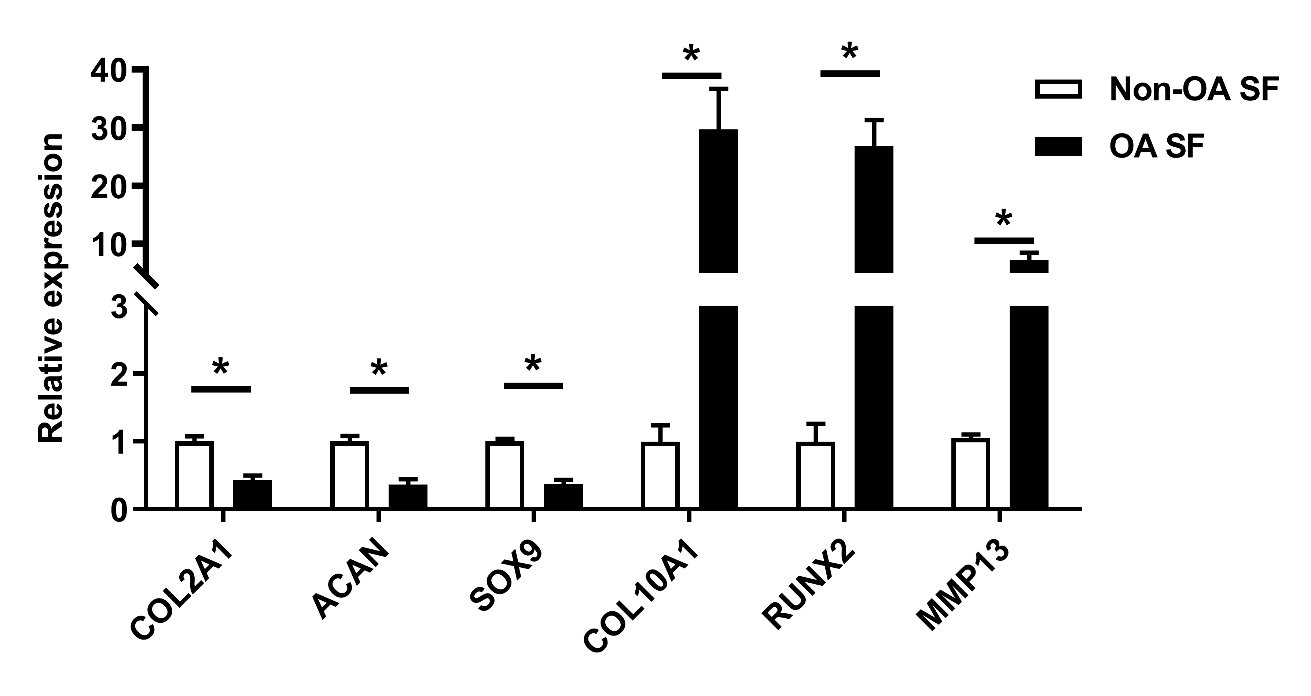

Supplement: Supplementary file 7 — Supplementary file7 [file 41598_2020_67446_MOESM7_ESM.docx]
