# Supplementary material for: SnoRNA signatures in cartilage ageing and osteoarthritis
Source: Sci Rep. 2020 Jun 30;10:10641. doi: 10.1038/s41598-020-67446-z (PMC7326970; doi:10.1038/s41598-020-67446-z)

**Supplementary File 8**. Effect of chondrocyte passage on snoRNA gene expression. There was little effect of passage on snoRNA expression. Passaged chondrocyte (n=5) snoRNA gene expression was relative to U6 and protein coding genes to GAPDH. Histogram represents mean ± standard error of mean. Statistical analyses undertaken using ANOVA.


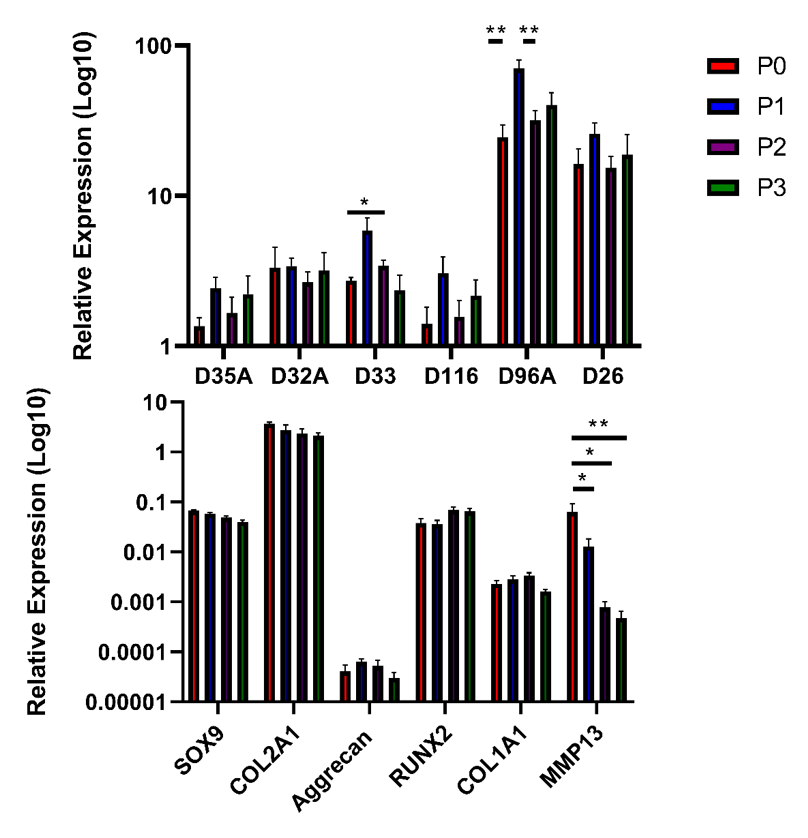

Supplement: Supplementary file 8 — Supplementary file8 [file 41598_2020_67446_MOESM8_ESM.docx]
