# Supplementary material for: SnoRNA signatures in cartilage ageing and osteoarthritis
Source: Sci Rep. 2020 Jun 30;10:10641. doi: 10.1038/s41598-020-67446-z (PMC7326970; doi:10.1038/s41598-020-67446-z)

Supplementary File 9. The effect of oxygen tension and serum in cell culture had no effect on selected snoRNA gene expression of non-OA HAC. A. P1 chondrocytes (n=3) were subject to 5% (low oxygen) and 20% (high oxygen) with (plus serum) and without (serum-free) 10% FCS. SnoRNA gene expression was relative to U6 and protein coding genes to GAPDH. Histogram represents mean ± standard error of mean. B. Represents assessment of chondrogenic and hypertrophic gene expression in the same samples. Statistical analyses undertaken using a one-way ANOVA.


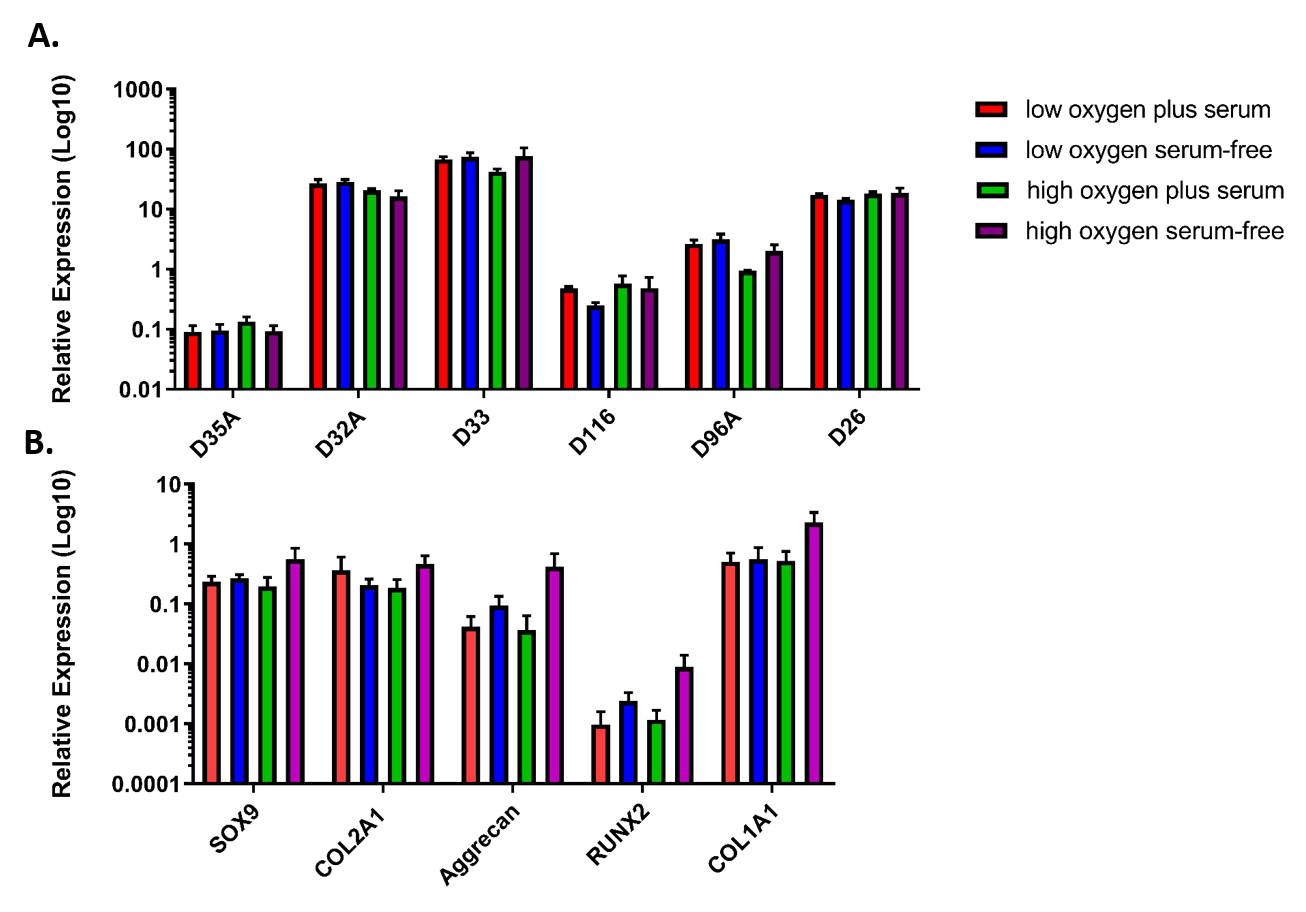

Supplement: Supplementary file 9 — Supplementary file9 [file 41598_2020_67446_MOESM9_ESM.docx]
